# Overestimating Resource Value and Its Effects on Fighting Decisions

**DOI:** 10.1371/journal.pone.0019924

**Published:** 2011-05-13

**Authors:** Lee Alan Dugatkin, Aaron David Dugatkin

**Affiliations:** 1 Department of Biology, University of Louisville, Louisville, Kentucky, United States of America; 2 Murray Hill Academy, Louisville, Kentucky, United States of America; University of Plymouth, United Kingdom

## Abstract

Much work in behavioral ecology has shown that animals fight over resources such as food, and that they make strategic decisions about when to engage in such fights. Here, we examine the evolution of one, heretofore unexamined, component of that strategic decision about whether to fight for a resource. We present the results of a computer simulation that examined the evolution of over- or underestimating the value of a resource (food) as a function of an individual's current hunger level. In our model, animals fought for food when they perceived their current food level to be below the mean for the environment. We considered seven strategies for estimating food value: 1) always underestimate food value, 2) always overestimate food value, 3) never over- or underestimate food value, 4) overestimate food value when hungry, 5) underestimate food value when hungry, 6) overestimate food value when relatively satiated, and 7) underestimate food value when relatively satiated. We first competed all seven strategies against each other when they began at approximately equal frequencies. In such a competition, two strategies–“always overestimate food value,” and “overestimate food value when hungry”–were very successful. We next competed each of these strategies against the default strategy of “never over- or underestimate,” when the default strategy was set at 99% of the population. Again, the strategies of “always overestimate food value” and “overestimate food value when hungry” fared well. Our results suggest that overestimating food value when deciding whether to fight should be favored by natural selection.

## Introduction

Behavioral ecologists have developed a suite of models for the evolution of fighting behavior [1]. Though different models of the evolution of aggression rely on different mathematical techniques and focus on different payoffs (mates, food, shelter and so on), they all share the following characteristic: at some point, animals assess both their relative chances of winning a fight, and the relative value of the resource being contested, when deciding whether or not to fight [2]–[7].

Two animals may assign different values to the same contested resource. It may be, for example, that a territory is more valuable to a territory holder (who has spent time learning about this area) than to an intruder [8]. Even in the absence of such an asymmetry (territory holder versus intruder), animals may still assign different values to the same resource. A hungry animal may, for example, value×units of food more than a satiated animal, and so be willing to fight longer or harder for that resource. Here, we explore the evolution of fighting strategies that involve “overestimating” or “underestimating” resource value as a function of current state [9], [10], and ask what conditions, if any, might favor the evolution of such strategies.

## Methods

### The Model

We used NetLogo simulation software to build an agent-based model [11]. A 53×53 torus (no edges) with 2,809 “cells” was used: each cell could hold one individual. The number of interactions each individual had with others in a given generation could be set from 1 to 10. The mean amount of food available per interaction was 50 food units, but the actual amount of food available for a given individual during a given interaction was randomly selected from a range of 0 to 100 food units. Individuals had no upper limit on the amount of resource they could take in, and there was no diminishing utility associated with the intake of additional resources.

An individual could use only one of seven strategies:

1. “Never over- or underestimate food value.” Individuals assessed the food value accurately.
2. “Always underestimate food value.” Individuals always underestimated the food value by some proportion (that could be set in the simulation from 0.01 to 0.99).
3. “Always overestimate food value.” Individuals always overestimated the food value by some proportion (that could be set in the simulation from 0.01 to 0.99).
4. “Overestimate food value when hungry.” Individuals overestimated the food value by some proportion (that could be set in the simulation from 0.01 to 0.99) when the units of food they had already obtained was less than 50 times the number of interactions (that is, less than the mean amount of food that would be expected after i interactions).
5. “Underestimate food value when hungry.” Individuals underestimated the food value by some proportion (that could be set in the simulation from 0.01 to 0.99) when the units of food they had already obtained was less than 50 times the number of interactions.
6. “Overestimate food value when (relatively) not hungry.” Individuals overestimated the food value by some proportion (that could be set in the simulation from 0.01 to 0.99) when the units of food they had already obtained was more than 50 times the number of interactions.
7. “Underestimate food value when (relatively) not hungry.” Individuals underestimated the food value by some proportion (that could be set in the simulation from 0.01 to 0.99) when the units of food they had already obtained was more than 50 times the number of interactions.

For all seven strategies examined, for the first interaction an individual had, we assumed that this individual had some amount of food in its gut already (the amount was a randomly selected number of food units from a range of 0–100).

Neighborhood size on the simulated grid was set at four individuals–the so-called “Moore neighborhood”–corresponding to the four slots that could be reached in a single move of a chess king [12]. At the start of a simulation, we began all seven strategies at equal frequencies (that summed to 1). Cells were then filled with individuals based on these initial frequencies (that is, each cell had a 1/7 chance of being filled with any of the seven different strategies).

The simulation then moved through each cell on the grid. The number of times this occurred in a single generation was simply the number of interactions individuals had per generation. Once an individual (let's call it individual 1) was selected, a randomly chosen opponent from its Moore neighborhood (individual 2) was simultaneously chosen. Each individual in such pairs used a simple rule as to whether to fight or not: fight when the estimate of a current food item is more than 50 units (the mean food value available on a given interaction). If both individual 1 and 2 decided to fight then, the outcome of a fight was determined as follows: at the start of a simulation, each individual was given a “fighting score” randomly selected from a range of fighting scores of 1–100. During interaction 1, the probability that individual 1 defeated individual 2 was:
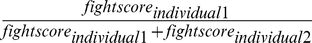
(1)Once a fight was decided, the fighting score of the winner was increased as follows:
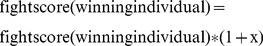
(2)where x could range from 0.01 to 1. The fighting score of the loser was decreased as follows:
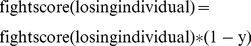
(3)where y could range from 0.01 to 1.

The winner obtained 75% of the food resource, and that amount was added to the food it had “in gut.” We assumed 75%, rather 100%, as a way to mimic the fact that fights take time, and that food resources may decline in value as a fight occurs. One way to think about this is that although winners always had a net gain in terms of resources, the 25% of the food not obtained by the winner was a kind of fighting cost. This cost is not constant (it is a function of the value of the resource), The amount of food in the loser's gut was decreased by some proportion (ranging from 0 to 0.75).

If one individual chose to fight, but the other individual chose not to fight, then the individual who was prepared to fight obtained 75% of the food resource. The amount of food in the gut of the individual who chose not to fight remained unchanged. The individual who opted to fight had its fight score increased exactly as in equation 3. The individual who opted to fight had its fighting score lowered as in equation 3. That is, fighting scores change the same way (i) whether an individual wins an actual fight or its opponent chooses not to fight, and (ii) whether an individual loses a fight or opts not to fight. Early runs of the simulation indicated that these assumptions did not alter our general results.

If both individuals in a pair chose not to fight, they split the amount of food (each receiving half), and neither of their fighting scores was changed.

At the end of all interactions in a given generation X, fitness was calculated as the amount of food in gut for all individuals of a given strategy. Generation X+1 was then seeded based on the relative fitnesses of strategies in generation X, and all other parameters were set back to those at the start of the first generation. As an example, if six strategies had the same fitness, but the seventh strategy had a fitness value twice that of the others, it would be represented at twice the frequency of these other six strategies in the subsequent generation. For each set of parameters chosen, we ran ten replicate simulations. We examined 36 different sets of starting parameters, and so ran a total of 360 initial simulations (these starting parameters are provided in the Supplementary Materials). These 36 sets of parameters varied in terms of the number of interactions per generation, the strength of overestimating or underestimating (strong to weak on each), and the extent to which fighting score was affected by winning or losing.

## Results

All runs of the simulation resulted in a single strategy reaching fixation–a frequency of 1–within 15,000 generations. In each and every one of these 360 scenarios, either “always overestimate food value” (269 times, 74.7%) or “overestimate food value when hungry” (91 times, 25.3%) reached fixation. In aggregate, these results suggest that overestimating food value, when such food is linked to victory in a fight, will often be favored by natural selection. When simulations were initiated with all seven strategies, we also found that: 1) increasing the number of interactions between individuals favored the evolution of the “always overestimate food value” over the “overestimate food value when hungry” strategy, and 2) increasing the amount by which food was overestimated favored the evolution of the “overestimate food value when hungry” over the “always overestimate food value” strategy 2.

Just because all runs of our simulation which began with the seven strategies in equal proportion ended with either “always overestimate food value” or “overestimate food value when hungry” going to fixation, does not mean that either of these strategies could necessarily invade a world that was composed primarily of “never over- or underestimate food value” (which we assume to be the ancestral state in a population). We next ran 36 simulations (each simulation repeated 10 times) in which we took whichever strategy–“always overestimate food value” or “overestimate food value when hungry”–that emerged from a given set of parameters (that is, that won the majority of the ten repetitions of a given set of parameters) in our initial simulation, and used those same parameters, except that “always overestimate food value” or “overestimate food value when hungry” was initiated at 1%, and “never over- or underestimate food value” was initiated at 99%. In all of these simulations, “always overestimate food value” or “overestimate food value when hungry” reached fixation (a frequency of 100%) in at least 3 of 10 replicates (range: 30 to 100% of 10 replicate runs ending in fixation for “always overestimate food value” or “overestimate food value when hungry”). In all simulations in which “always overestimate food value” or “overestimate food value when hungry” did not reach fixation, they went to a frequency of the zero.

## Discussion

We used an individual-based computer simulation to investigate the evolution of over- or underestimating the value of a resource (food) as a function of an individual's current hunger level. Given the assumptions we outline in the model section, our computer simulations found that strategies that overestimate food resources–either when hungry, or always–should be favored by natural selection. Conversely, our simulations found that underestimating the value of food resources was never a good strategy. This later result is not necessarily intuitive–it might have been that animals that already had lots of food in their gut would be favored to underestimate food resources, and hence fight for them less often. Why underestimating a food resource was never favored by selection in our model deserves further investigation.

It might be argued that overestimating food resources is only favored by selection in our model because it makes animals more likely to engage in contests for food. We do not disagree with such an interpretation. After all, in *any* model for the evolution of fighting, *some variable*(s) is responsible for influencing the decision to fight or not. We simply note here that the estimation of a food item's value is one such variable. That said, we note that the loser of a fight incurred costs, and that when two individuals both opted not to fight, each could get resources, so a priori, it did not *have* to be the case that overestimating, and fighting more often as a result, was necessarily a better strategy in our model.

We know of no other specific models in behavioral ecology that address the questions we raise here, nor do we know of any studies in behavioral ecology that have been designed to examine under- and over-estimation strategies. But, we encourage empiricists to develop methods for quantifying the extent to which animals overestimate the value of food resources. Once such methods have been developed, the extent to which animals use overestimation strategies can be examined. Equally important, fine-tuning of such methods will allow a test of the predictions of our model–for example, one could test our prediction that increasing the number of interactions between individuals favors the evolution of the “always overestimate food value” over the “overestimate food value when hungry” strategy, and a comparative analysis could test our prediction that greater degrees of overestimation favors the “always overestimate food value” over the “overestimate food value when hungry” strategy.

Finally, we note that while our model used food as the resource contested, our results are more general than this, as they apply to any resource whose value can be estimated by animals.
